# Location and access to health courses for rural students: an Australian audit

**DOI:** 10.1186/s12909-024-05787-3

**Published:** 2024-07-29

**Authors:** Carol McKinstry, Claire Quilliam, Nicole Crawford, Jason Thompson, Stephanie Millns Sizer

**Affiliations:** 1 La Trobe Rural Health School, La Trobe University, Edwards Rd, Flora Hill, VIC 3552 Australia; 2 Violet Vines Marshman Centre of Rural Health Research, La Trobe University, Edwards Rd, Flora Hill, VIC 3552 Australia; 3 The University of Melbourne, 49 Graham Street, Shepparton, VIC 3630 Australia; 4 Curtin University, Kent Street, Bentley, WA 6102 Australia; 5 Centre for Research in Assessment and Digital Learning, Deakin University, 727 Collins Street, Melbourne, 3008 Australia; 6 Melbourne School of Design, University of Melbourne, Parkville, VIC 3010 Australia; 7 Geraldton Universities Centre, 33 Onslow St, Geraldton, WA 6530 Australia

**Keywords:** Higher education, Student equity, Health courses, Rural workforce, Access, Nursing, Allied health, Medicine, Dental

## Abstract

**Background:**

The undersupply of health professionals in rural areas impacts healthcare access for those living in rural Australia. A strategy to increase the rural health workforce is to recruit and educate rural people. However, long-standing inequities for rural Australians in accessing tertiary education persist. The aim of this study was to audit the 2023 offerings of Australian allied health, nursing, dental and medical university courses to identify geographical availability and those delivered online.

**Methods:**

A desktop audit of Australian allied health, nursing, dental and medical courses offered in 2023 was undertaken to identify the courses and delivery modes of those courses offered in regional, rural and remote locations. The audit involved searching lists of professionally accredited courses and university websites, which is publicly available information about health courses. Data were tabulated and descriptive statistics used for data analysis.

**Results:**

There were marked differences in online and rural course offerings across health professions in Modified Monash (MM) Model category 2–7 locations. Nursing/midwifery had the most courses while pharmacy, podiatry, dental and medicine had few offerings and optometry had none. Social work, nursing/midwifery and psychology also had the most online course offerings. Most courses were offered in MM2 and MM3 locations with few offerings in rural or remote areas. The availability of studying part-time was very limited and often this was only for the early years of the course. Inconsistencies relating to the course information on university websites existed relating to course delivery mode descriptions.

**Conclusions:**

There is a lack of rural on-campus or online course offerings for some allied health disciplines, dentistry and medicine. Provision of end-to-end, flexible courses in rural areas or online is needed to reduce access barriers for rural students and to enable sustainable rural health workforce development.

**Supplementary Information:**

The online version contains supplementary material available at 10.1186/s12909-024-05787-3.

## Background

Providing higher education health courses closer to or within rural areas is key to strengthening rural health workforces (WHO) [1]. In Australia and elsewhere, a higher proportion of health professionals reside in metropolitan centres than in regional, rural or remote areas (herein referred to as rural areas) compared with the average population [2]. The undersupply of health professionals in rural areas prevents timely access to healthcare for rural people [3] who often have greater needs for health services due to increased incidences of chronic disease and ageing populations [4, 5].

Historically, most Australian universities delivering health professional education have been located in metropolitan cities with fewer courses offered on regional or rural university campuses or online [6]. Reports dating back to the Bradley Review of Australian Higher Education “Transforming Australia’s Higher Education System” in 2008 [7] and the Napthine Review into Australia’s Regional, Rural and Remote Higher Tertiary Education Strategy [8] have highlighted the inequity issues relating to access for rural and regional students to tertiary education. The recent Australian Universities Accord Final Report [9] has also made bold recommendations and suggested strategies to lift tertiary education participation rates across Australia, particularly for rural populations. The Australian Government Productivity Commission in 2019 found that although there had been significant expansion in providing higher education particularly to those students from lower socio-economic status backgrounds, there had been minimal improvement for groups such as Indigenous people and those living in regional, rural or remote locations [10]. The limited access to tertiary education for rural people is negatively affecting rural health workforce issues in Australia including recruitment and retention of rural health professionals.

Characteristics of students accessing tertiary education in rural areas is different to those in cities, which means that the delivery of health courses by universities in rural areas requires more nuanced planning. Analyses of the 2018 and 2021 national higher education student participation data shows that – for domestic undergraduate student populations – there are higher proportions of mature-aged students in regional and remote areas compared to metropolitan areas, and this increases with remoteness [11–13]. Compared with their metropolitan counterparts, the proportion of students from low socio-economic status (SES) areas was also higher in the regional and remote areas [12]. Furthermore, the proportion of students who were female, Aboriginal and Torres Strait Islander, and who studied online and part-time also increased with remoteness [12]. Many mature-aged students in regional, rural and remote areas do not relocate for their studies due to existing family and work commitments in their local community [12]. Considering these multiple factors, additional and specific student supports need to be provided by universities to attract and retain students. Fewer prospective students, lower participation rates in higher education by those in rural areas and less access to vocational education and training courses results in ‘thin markets’ for university providers in rural areas [14].

Cuts to Australian higher education budgets have placed university providers under increased pressure to deliver financially sustainable courses and discontinue unprofitable ones [14]. Even minor student withdrawal rates can jeopardise the financial viability of rural campus courses, making universities apprehensive to provide health courses in rural areas. Despite evident health workforce demands, little is known about the current rural health course offerings in rural areas in Australia. Having a better understanding of rural health course availability will allow policymakers to work in partnership with other stakeholders, including universities, to better meet the educational needs of rural people and address health workforce demands.

The aim of this study was to audit Australian allied health, nursing, dental and medical university courses delivered in regional, rural and remote Australia, including the availability of online courses. The specific research question for the study was: What Australian allied health, nursing, dental and medical university courses are offered in regional, rural and remote locations, and online, and how are they delivered?

## Methods

A desktop audit of Australian allied health, nursing, dental and medical university courses offered in 2023 was undertaken in 2022. Modified Monash (MM) Model classifications [15] were used to determine rurality of the courses. The MM has seven classifications ranging from metropolitan (MM1) to very remote areas (MM7) [15]. In this study, we focused on the MM2 to MM7 classifications including regional centres (MM2); large, medium and small rural towns (MM3, MM4, MM5); remote (MM6); and very remote communities (MM7). Metropolitan areas (MM1) were excluded. Audited courses were those listed as approved on the official websites of peak bodies of health disciplines or registration boards. These disciplines were identified as having rural health workforce maldistribution and in demand through recent policy development, including the National Disability Insurance Scheme [16]. They included: optometry, pharmacy, occupational therapy, physiotherapy, podiatry, psychology, social work, speech pathology nursing, dental and medical.

A desktop audit of the Australian Health Practitioner Regulation Agency (AHPRA) website was first undertaken to identify professional accredited courses, as well as eligibility requirements for Australian graduate registration for all included AHPRA health disciplines. The websites of the peak associations including the Australian Association of Social Work and Speech Pathology Australia were also audited for accredited courses. Only pre-professional courses that enabled Australian professional registration were included in the audit, meaning postgraduate courses that required students to already be qualified in that discipline-specific health profession were excluded. To ensure that the courses listed on these websites were still being offered, the course information on individual university websites were checked in 2021 and 2022, so that 2023 course offerings and course details were accurate. Human research ethics approval was not required because all data used were publicly available and did not involve data regarding study participants.

The name of the course, the campus location, study type (full-time, part-time), and study mode (on-campus, online, multi-modal) were recorded which enabled understanding of access gaps and the extent of those gaps across disciplines. The opportunity to complete an honours qualification was noted but not viewed as a separate course. Researcher CM conducted the initial audit of the AHPRA or professional association website and then members of the research team (CM, CQ or NC) checked the university websites to ensure that the courses were still being offered for 2023. When it was identified that a course may not be offered, this was discussed by the research team and validated, for example, via email correspondence with course providers.

Additional attention was paid to psychology courses offerings because unlike other health professions, there are a number of pathways to become an AHPRA registered psychologist. According to the AHPRA website (https://www.psychologyboard.gov.au/Registration/General.aspx), a six-year sequence of education is required. The higher degree pathway involves studying a four-year accredited course and then a further two years (fifth and sixth year of the six-year sequence), undertaking a postgraduate degree such as a two-year masters, or a doctorate involving three-four years of study. Another pathway to registration is the five-plus-one-year internship pathway, where five years of study is undertaken via a Master of Professional Psychology and a further one year internship involving intensive supervision. The pathway involving four years of study in an accredited psychology course (usually an honours degree or equivalent) and a two-year internship was being phased out with no further applications accepted after 30th June 2022.

Provisional registration is provided to those undertaking an internship. Given the complexity of options relating to psychology registration, the mapping of pathway degrees was not straight forward. The array of pathway four-year degrees as part of the six-year sequence of education is substantial. Students can study a Bachelor of Arts or a Bachelor of Science, majoring in psychology, or study a double degree linking to a Bachelor of Arts or Science or Psychology with another area such as commerce, exercise science or criminology. In our mapping we sought to capture potential pathway degrees offered by universities online or at campuses outside of MM1 locations that were listed on the AHPRA website and verified these against university website information. Courses such as a Master of Clinical Psychology, Master of Psychology or Master of Professional Psychology were also included given these are needed for the first pathway to registration.

When attempting to determine course mode, we used a classification consistent with the course reporting of Australian universities to the Australian Government Department of Education which included internal, external, and multi-modal modes of attendance [17]. ‘Internal’ mode of attendance referred to courses that were “undertaken through attending at a higher education provider on a regular basis” or “where students attend on an agreed schedule for the purposes of supervision and/or instruction”, otherwise described as face-to-face classes and on campus attendance [17] (Australian Government Tertiary Collection of Student Information, n.d.). ‘External’ mode of attendance is described as having lesson materials, assessments, etc., being delivered to the student or where other attendance at the university was “incidental, irregular or for a special reason or voluntary nature” (Australian Government Tertiary Collection of Student Information, n.d.). External mode of attendance also refers to online, distance, and off-campus course delivery. Multi-modal attendance involves students studying partially on campus as “internal mode of attendance and partially as external mode of attendance” [17].

A spreadsheet was used to collect and organise the data according to discipline. Data were tabulated and analysed using descriptive statistics, and then converted to a map using ArcGIS, a web-based mapping software (ArcGIS Online, https://www.arcgis.com/index.html) to provide a visual representation of the geographical locations of campuses where health courses were delivered in MM2-MM7 classified areas.

## Results

Results showed that 259 pre-professional health courses were available in MM2-7 locations and online in the allied health, nursing, dental and medical disciplines for the 2023 intake (see Table 1). Approximately one-third (32%) were delivered online, with the majority of these being psychology courses. Forty per cent were delivered in regional centres (MM2 locations), with the majority of these being nursing and midwifery courses. Twenty-five per cent of all courses were delivered in large rural towns (MM3 locations), with the majority of these being nursing and midwifery courses. Psychology offered the most courses in MM2-7 locations and online. Optometry was the only discipline to not offer a course in any MM2-7 locations or online. Table 1 illustrates the dearth of health courses available as remoteness increased, with gaps evident in many columns, particularly in medium and small rural towns (MM4 and MM5) and remote and very remote communities (MM6 and MM7).

**Table 1:** Australian Allied Health, nursing, Dental and Medical courses, including MM locations

Discipline	MM2	MM3	MM4	MM5	MM6	MM7	Online	Total
Optometry								0
Podiatry	2							2
Dentistry	2							2
Oral Health	3							3
Medicine	2	3						6
Pharmacy	5	1						6
Speech Pathology	7	2						9
Occupational Therapy	7	3						10
Physiotherapy	10	2						12
Social Work	8	7					8	23
Nursing and Midwifery	29	25	2	3		1	12	72
Psychology	28	22					64	114
Total	104	65	2	3		1	84	259

**Fig. 1:**
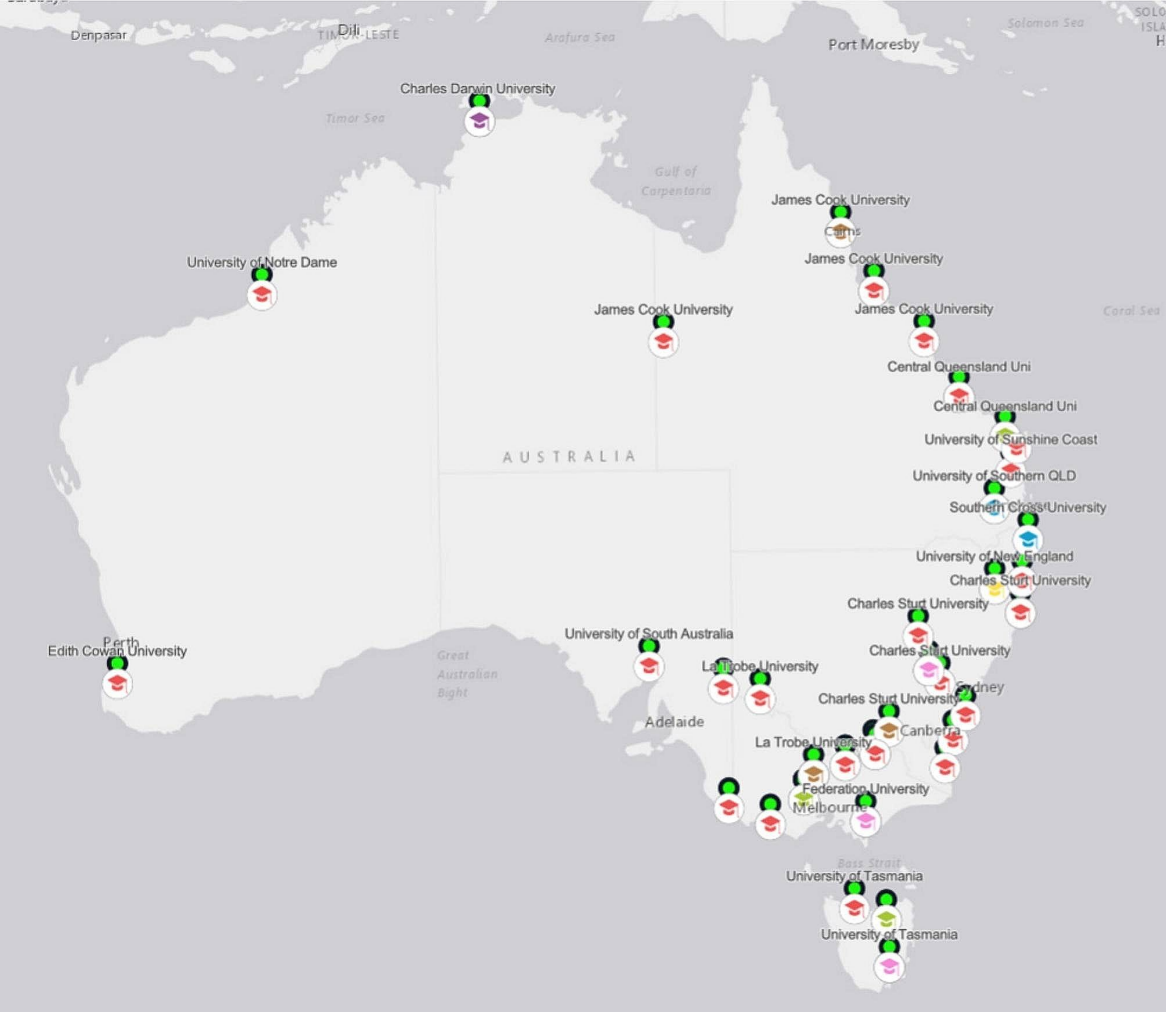
Mapping of university campuses with health courses in MM2-7 locations

As illustrated in Fig. 1, when examining the geographical locations of pre-professional courses across Australia, very few courses are located in regional, rural or remote areas of Western Australia, South Australia and Northern Territory. For interactive maps providing information compiled from our audit such as the location of individual professional courses, MM2-7 locations, university campus locations, and local government areas, refer to https://experience.arcgis.com/experience/64fb93f6fe074035ba3f3314454c1744.

The findings related to individual discipline course access are provided in an ascending order, starting with the discipline with the least number of course offerings to those with the most. Most disciplines are presented in individual tables. However, disciplines such as nursing, psychology, and social work, where offerings were extensive, are available as an additional file in the Supplementary Information. Pertinent findings are also described for each discipline.

### Optometry

No optometry courses offered outside of MM1 locations. It was also the case that no optometry courses were delivered online.

### Podiatry

Only two courses were offered outside an MM1 location, with a Bachelor of Podiatry offered at the Rockhampton campus of Central Queensland University in Queensland and a Bachelor of Podiatric Medicine offered by Charles Sturt University at the Albury campus in New South Wales (see Table 2). Both courses were in MM2 locations and offered some flexibility to study, with Central Queensland University offering a number of subjects via distance which were supplemented with face-to-face residential schools. Charles Sturt University’s website indicated that it was an on-campus course; however, some subjects could be studied online with residential school attendance required.

**Table 2:** Podiatry courses according to MM location, university, and mode of delivery

MM	Course	University/Location	Mode of Delivery	Additional Details
MM2	Bachelor of Podiatry*	Central Queensland University / Rockhampton	Internal	Some subjects delivered via distance and residential schools.
	Bachelor of Podiatric Medicine*	Charles Sturt University / Albury	Internal	Residential blocks required.

No courses in MM3 -MM7 or online were listed on the AHPRA website.

*Honours also offered

### Dental / oral health

Only three oral health courses were offered outside of MM1 areas and these were in MM2 locations (see Table 3). The three oral health courses were in Rockhampton (Queensland), Wagga Wagga (New South Wales), and Bendigo (Victoria). For dentistry courses, the three locations were Cairns (Queensland), Orange (New South Wales), and Bendigo (Victoria). According to university websites, the dentistry course at James Cook University in Cairns could be studied part-time for the first three semesters. The Charles Sturt University website suggested the dentistry course in Orange is offered on-campus however part-time study mode was a possibility, along with some online subjects that required residential school attendance.

**Table 3:** Dental and oral health courses according to MM location, university, and mode of delivery

MMM	Course	University/Location	Mode of Delivery	Additional Details
MM2	Bachelor of Oral Health	Central Queensland University / Rockhampton	Internal	
	Bachelor of Oral Health (Therapy and Hygiene)	Charles Sturt University / Wagga Wagga	Internal	Residentials mentioned.
	Bachelor of Oral Health Science	La Trobe University / Bendigo	Internal	
	Bachelor of Dental Science	Charles Sturt University / Orange	Internal	Residentials mentioned.
	Bachelor of Dental Surgery*	James Cook University / Cairns	Internal	Part-time available year 1 and semester 1 year 2.
	Bachelor of Dental Science*	La Trobe University / Bendigo	Internal	

No courses in MM3 -MM7 or online were listed on the AHPRA website.

*Honours also offered

### Medicine

A total of six courses were offered outside of MM1 areas, with three courses offered in MM2 and three in MM3 locations (see Table 4). The courses in MM2 locations were at Orange (New South Wales), Hobart (Tasmania) and Townsville (Queensland). The courses in MM3 locations were at Churchill (Victoria), Shepparton (Victoria), and Armidale (New South Wales).

**Table 4:** Medicine courses according to MM location, university, and mode of delivery

MM	Course	University/Location	Mode of Delivery	Additional Details
MM2	Doctor of Medicine	Charles Sturt University / Orange	Internal	Partnership with Western Sydney University.
	Bachelor of Medicine / Bach of Surgery	James Cook University / Townsville	Internal	
	Bachelor of Medicine & Bachelor of Surgery	University of Tasmania / Hobart	Internal	
MM3	Bachelor of Medical Science / Doctor of Medicine (MD)	Monash University/ Churchill	Internal	Graduate entry students study the first year at Churchill and then the following 3 years in Melbourne, rural south-east Victoria and rural north-west Victoria.
		University of Newcastle & University of New England / Armidale	Internal	Joint program between the two universities.
	Doctor of Medicine	University of Melbourne / Shepparton	Internal	

No courses in MM4 -MM7 or online were listed on the AHPRA website.

### Pharmacy

Six universities offered pharmacy courses outside of an MM1 location (see Table 5). These were offered in Bendigo (Victoria), Orange (New South Wales), Hobart (Tasmania), Townsville, Mackay, and Cairns (Queensland), and Darwin (Northern Territory). No courses were offered in regional or rural areas in Western Australia or South Australia. All courses were Bachelor of Pharmacy including honours offerings and only one course was offered at a campus in an MM3 location, which was at Armidale (New South Wales). According to university websites, part-time study was possible for courses at five locations, especially in the earlier years of the course.

**Table 5:** Pharmacy courses according to MM location, university, and mode of delivery

MM	Course	University/Location	Mode of Delivery	Additional Details
MM2	Bachelor of Pharmacy*	Charles Sturt University / Orange	Internal	Residential schools
		Charles Darwin University / Darwin		Students can study for a maximum of 8 years suggesting part-time study is available.
		James Cook University / Townsville	Internal	Part-time only available for years 1 and 2
		James Cook University / Mackay	Internal	Part-time only available for years 1 and 2
		James Cook University / Cairns	Internal	Part-time only available for years 1 and 2
		La Trobe University / Bendigo	Internal	
	Bachelor of Pharmacy with Applied Honours	University of Tasmania / Hobart	Internal	Students can take a maximum of 9 years of study suggesting part-time study is available.
MM3	Bachelor of Pharmacy	University of New England / Armidale	Internal	Online with intensive schools as well as on-campus classes

No courses in MM4 -MM7 or online were listed on the AHPRA website.

*Honours also offered

### Speech pathology

A total of nine speech pathology courses were offered outside of MM1 locations. These were Bachelor of Speech Pathology courses including honours courses (*n* = 4), graduate entry Master of Speech Pathology courses (*n* = 4) and a double degree (Bachelor of Health Science and Master of Speech and Language Therapy) (see Table 6). Seven courses were in MM2 locations in the eastern states of Australian and in the Northern Territory. There were two courses in MM3 locations which included a course in Coffs Harbour (New South Wales) and a course in Gippsland (Victoria). Most of the courses were delivered on campus; however, Federation University’s Master of Speech Pathology at the Churchill and Mount Helen campuses (Victoria) were delivered on campus and via flexible delivery or multi-modal. The Bachelor of Speech Pathology at James Cook University in Townsville (Queensland) indicated that some flexibility regarding part-time study during the first two years of study. The website of the Master of Speech Pathology at University of Tasmania in Launceston (Tasmania), which commenced in 2023, indicated that students could take a maximum of five years to complete the course, suggesting the possibility of part-time study.

**Table 6:** Speech Pathology courses according to MM location, university, and mode of delivery

MM	Course	University/Location	Mode of Delivery	Additional Details
MM2	Bachelor of Speech Pathology*	Central Queensland University / Rockhampton	Internal	
		James Cook University / Townsville	Internal	Part-time available in the first 2 years.
		La Trobe University / Bendigo	Internal	
	Master of Speech Pathology	La Trobe University / Bendigo	Internal	
		University of Tasmania / Launceston	Internal	Students can take up to 5 years to complete, suggesting part-time study is available.
		Federation University / Mount Helen	Internal	
		Federation University / Mount Helen	Multi-modal	Flexible delivery.
	Master of Speech and Language Therapy	Charles Darwin University / Darwin	Multi-modal	2 years full-time or 4 years part-time.
MM3	Bachelor of Speech Pathology	Southern Cross University / Coffs Harbour	Internal	
	Master of Speech Pathology	Federation University / Churchill	Internal	
		Federation University / Churchill	Multi-modal	Flexible delivery.

No courses in MM4 -MM7 or online were listed on the Speech Pathology Australia website.

*Honours also offered

### Occupational therapy

Occupational therapy courses offered at locations outside MM1 locations included Bachelor of Occupational Therapy degrees, graduate entry Masters and one double degree (Bachelor/Master) in MM2 and MM3 locations (see Table 7). In MM3 locations, three Bachelor of Occupational Therapy courses (with and without honours) were offered in locations within Victoria and New South Wales. Of the courses offered in MM2 locations, none were offered in Western Australia or South Australia. It was difficult to determine if courses were available via part-time; however, the James Cook University’s website stated that part-time study during the first two years of the course was available, while the courses at Charles Darwin University could be undertaken part-time. The courses at Charles Darwin University were also listed as being offered on-campus and online.

**Table 7:** Occupational therapy courses according to MM location, university, and mode of delivery

MM	Course	University/Location	Mode of Delivery	Additional Details
MM2	Bachelor of Occupational Therapy*	Central Queensland University / Bundaberg	Internal	Some subjects can be studied via distance education but may need to attend residential classes.
		Central Queensland University / Rockhampton	Internal	Some subjects can be studied via distance education but may need to attend residential classes.
		Charles Sturt University / Albury-Wodonga	Internal	Requirement for students to attend residential schools for some online units.
		James Cook University / Townsville	Internal	
		La Trobe University / Bendigo	Internal	
	Bachelor of Health Science/Master of Occupational Therapy	Charles Darwin University / Darwin	Internal Online	4 years full-time or 8 years part-time
	Master of Occupational Therapy	Charles Darwin University / Darwin	Internal Online	2 years full-time or 4 years part-time
MM3	Bachelor of Occupational Therapy*	Charles Sturt University / Port Macquarie	Internal	Requirement for students to attend residential schools for some online units.
		Federation University / Churchill	Internal	
		Southern Cross University/ Coffs Harbour	Internal	Face-to-face contact is listed as the teaching method on the university website.

No courses in MM4 -MM7 or online were listed on the AHPRA website.

*Honours also offered

### Physiotherapy

Seven Bachelor of Physiotherapy courses were offered, which included an honours cohort in MM2 locations and two in MM3 locations (see Table 8), on campuses in Victoria, Queensland and New South Wales. There were also two graduate entry Masters courses offered in MM2 locations in Victoria and Tasmania, and a double degree (Bachelor of Health Science and Master of Physiotherapy) in Darwin, Northern Territory. The relatively new Masters course offered in Tasmania was described as being delivered flexibly, including intensive practical blocks. The double degree offered in Darwin was advertised as being available for part-time study.

**Table 8:** Physiotherapy courses according to MM location, university, and mode of delivery

MM	Course	University/Location	Mode of Delivery	Additional Details
MM2	Bachelor of Physiotherapy*	Australian Catholic University / Ballarat	Internal	
		Central Queensland University / Bundaberg	Internal	
		Central Queensland University / Rockhampton	Internal	
		Charles Sturt University / Albury	Internal	Requirement for students to attend residential schools for some online units.
		Charles Sturt University / Orange	Internal	Requirement for students to attend residential schools for some online units.
		James Cook University / Townsville	Internal	
		La Trobe University / Bendigo	Internal	
	Master of Physiotherapy Practice	La Trobe University / Bendigo	Internal	
	Master of Physiotherapy	University of Tasmania / Launceston	Internal	Flexible delivery including intensive practical blocks
MM3	Bach of Physiotherapy*	Charles Sturt University / Port Macquarie	Internal	Requirement for students to attend residential schools for some online units
		Federation University / Churchill	Internal	

No courses in MM4 -MM7 or online were listed on the AHPRA website.

*Honours also offered

### Social work

Social work courses offered outside of MM1 areas were in MM2 and MM3 locations (see Table 9). Eight social work courses were in MM2 locations, with three Bachelor degrees and five graduate entry master degrees. Courses were located in Northern Territory, Queensland and Victoria. In MM3 locations, there were six Bachelor degrees and one graduate entry Masters degree. These courses were mostly in New South Wales with two in South Australia. Most courses indicated that a part-time study option was available. Most courses in MM3 locations had a combination of online and residential school delivery of teaching. Eight online courses were available with five being Bachelor degrees and three graduated entry Masters degrees. These were offered by universities in New South Wales, Queensland, Northern Territory and Victoria.

**Table 9:** Social work courses according to MM location, university, and mode of delivery

MM	Course	University/Location	Mode of Delivery	Additional Details
MM2	Bachelor of Social Work*	Charles Darwin University / Darwin	Multi-modal	Part-time available
		James Cook University / Townsville	Internal	Part-time available
		La Trobe University / Bendigo	Internal	Part-time available
	Master of Social Work	Charles Darwin University / Darwin	Internal	Part-time available
		James Cook University / Townsville	Internal	Part-time available
		James Cook University / Cairns	Internal	Part-time available
		La Trobe University / Bendigo	Internal	Part-time available
		Federation University / Ballarat		
MM3	Bachelor of Social Work	Charles Sturt University / Dubbo	Internal	On-campus and online with residential schools
		Charles Sturt University / Port Macquarie	Internal	On-campus and online with residential schools
		Charles Sturt University / Wagga Wagga	Internal	On-campus and online with residential schools
		University of New England / Armidale	Internal	
		University of South Australia / Mount Gambier	Multi-modal	Part-time available
		University of South Australia / Whyalla	Multi-modal	Part-time available
	Master of Social Work	University of New England / Armidale	Internal	Professional qualifying.
No courses in MM4 -MM7 were listed on the Australian Association of Social Workers website.				
Online	Bachelor of Social Work	Charles Sturt University	External	
		University of New England	External	
		Charles Darwin University	External	
		Central Queensland University	External	
		Deakin University	External	Online, distance education
	Master of Social Work	Charles Sturt University	External	Professional qualifying.
		University of New England	External	Professional qualifying.
		Charles Darwin University	External	

*Honours also offered

### Nursing and midwifery

A wide range of nursing course offerings, particularly in MM2 locations, were available for students entering directly from secondary schooling, as enrolled nurses or those already having a university degree (see Table 1 in the Additional Information File). Most of these courses were Bachelor of Nursing or Bachelor of Science (Nursing). There was one Bachelor of Midwifery and three double degrees in nursing and midwifery. At Charles Darwin University’s Darwin campus, a Diploma of Health Care/Bachelor of Nursing was offered. Two Bachelor of Nursing graduate entry degrees in Victoria and New South Wales, and a graduate entry Master of Nursing Practice at Charles Darwin University in the Northern Territory were on offer. A double degree in nursing and paramedicine was offered at Federation University in Victoria in an MM2 location.

In MM3 locations, there was again a high number of Bachelor of Nursing (with and without honours) offered, particularly in South Australia, Tasmania, New South Wales and Victoria. Bachelor of Nursing degrees were offered in Victoria and New South Wales for enrolled nurses while only two graduate entry Bachelor of Nursing degrees were offered, with both being in Victoria. Only one Bachelor of Midwifery course was offered.

Only two nursing and midwifery courses were offered in MM4 locations, three courses offered in MM5 locations, and one course offered in an MM7 location. No nursing or midwifery courses were in MM6 locations, while no courses in midwifery were offered in MM4-7. There was also a range of courses offered as online or distance education although requiring mandatory on-campus intensive schools or residential schools. Overall, many nursing courses offered a part-time study option (See Table 1 in the Additional Information File). As Table 1 illustrates, 75 per cent of all nursing courses offered were in MM2-3 locations, meaning eight per cent of courses were available in MM4-7 locations, and 16 per cent online.

### Psychology

Offerings by universities in MM2 locations for psychology were vast with possible pathway Bachelor or double degree courses available in Victoria (*n* = 4), Queensland (*n* = 8), Northern Territory (*n* = 2) and Tasmania (*n* = 12) (see Table 2 in the Supplementary Information file). Two postgraduate degrees were offered by the University of Tasmania. Of the courses offered in MM3 locations, there were undergraduate courses, including double degrees in Victoria (*n* = 4) and New South Wales (*n* = 14), and three postgraduate degrees in New South Wales. The online offerings of psychology courses were extensive with over seventy found. Twenty-six postgraduate courses were identified; however, the vast majority of these were graduate diplomas of psychology and it was difficult to determine if they enabled articulation into other courses that may fulfil AHPRA registration requirements.

### Terminology

The Australian Department of Education requires universities to report the mode of attendance for each course to support students to make decisions about future study. The Tertiary Education Quality and Standards Agency (TEQSA) in Australia has a list of different course delivery modes; however, there are no definitions provided or agreed upon definitions existing in the sector (see https://www.teqsa.gov.au/guides-resources/glossary-terms#modestudy ). The audit revealed inconsistencies in course information on university websites, particularly related to the terminology used to describe the external mode of delivery.

## Discussion

This study explored the availability of Australian allied health, nursing, dental and medical university courses in regional, rural and remote areas for 2023 by conducting a desktop audit and using the MM classifications to categorise course location and identify courses delivered online. The findings illustrate relatively few options to study health courses outside metropolitan areas, particularly in medium and small rural towns (MM4 and MM5), and remote and very remote communities (MM6 and MM7). For example, in some disciplines, such as optometry, there are no courses in MM2-7 locations, no courses in MM3-7 locations for podiatry, dentistry and oral health, and only one pharmacy course in an MM3 location. These findings indicate that despite significant effort to develop the rural health workforce [2], the fundamental strategy of providing higher education health courses in rural areas has been overlooked at the same time as it is being recommended as good practice in the World Health Organization’s [1] guidelines on health workforce development. The findings also identify equity issues for people living in regional, rural and remote areas who cannot afford or are not able to move to metropolitan areas to study specific health professions, particularly those not living in eastern Australian states or territories.

The lack of higher education health course offerings in some disciplines accessible in or from rural areas highlighted in this study has implications for the Australian rural health workforce and access to healthcare for rural communities. Having no optometry courses being provided in rural areas may be contributing to the long-standing maldistribution of the optometry workforce [18]. Kirkman and colleagues [19] suggested the rural pipeline approach, involving recruitment of rural health students and educating in a rural location, is needed to resolve the maldistribution for optometry. In other disciplines, such as speech pathology, occupational therapy, physiotherapy and podiatry, relatively few courses are offered in rural areas beyond MM3 communities. The lack of course availability in these disciplines is consistent with workforce maldistribution and further exacerbates the impact of the significant increase in demand for services in these disciplines due to the introduction of the Australian National Disability Insurance Scheme (NDIS) [16] and an ageing population particularly in rural areas [20].

Since the NDIS was introduced, demand for speech pathology services has increased [21]. Similarly, occupational therapy is the fastest growing registered health profession in Australia and is still struggling to meet service demand [22]. Significant delays in access to services offered by these professions are commonly experienced in rural and regional areas, affecting participation in daily life and continuity of care. Demand for speech pathology and occupational therapy services in rural areas is not expected to decrease, given increases in ageing populations [23]. The paucity of rural health professionals also contributes to overwork and burnout among those who remain [24–26]. Innovative solutions are required to ensure health course offerings are made more accessible to rural people to help address longstanding issues around workforce demands and timely access to healthcare for patients in rural areas.

Universities encounter challenges when offering health courses on regional and rural campuses. Disciplines such as dentistry, oral health, optometry and podiatry have high course operating expenses due to the equipment and facilities required for simulation and student clinical skill development in preparation for placements. Challenges attracting academic workforce and clinical supervisors may be experienced particularly for dental and oral health courses in regional areas [27]. While the Rural Health Multidisciplinary Training program provides funding to support Rural Clinical Schools and Universities Departments of Rural Health to increase the rural workforce, an evaluation of the program in 2023 highlighted that most clinical placements were occurring in large regional settings and less in smaller rural communities [28]. The evaluation report recommended that longer periods of rural immersion for allied health and nursing students was needed which is less relevant for universities with end-to-end courses on regional or rural campuses where placements are predominantly located in rural areas and a greater percentage of students have rural origins.

The limited opportunity for people living in rural areas to study locally or remotely is problematic and exacerbates an existing equity issue. Relocating to metropolitan centres to study poses a significant barrier to entry, particularly for mature-aged students who may have family, carer, and work commitments [12, 29]. These are in addition to significant social and financial costs for individual school-leavers and their families [29, 30]. While studying online can provide access to higher education for students who otherwise could not participate, it can also pose limitations relating to the quality and experience of online learning [31, 32], internet connectivity [33] and reduce the sense of belonging and peer support crucial for completing high intensity study, especially for mature-aged students [34, 35].

As findings here illustrated, many health disciplines do not deliver courses online or even flexibly via intensive on-campus or residential block attendance. Hence, for most disciplines except for nursing, social work and psychology, online study is not an option for Australian rural students. More flexible delivery of courses, such as online and/or part-time study may increase the likelihood of rural students being able to participate in health courses while remaining in their rural communities. Such delivery is important for mature-aged students in rural areas (who are unlikely to re-locate for studies) and is crucial for building the rural health workforce capacity of people who are committed to staying in and contributing to their communities. Studying online can also enable students to manage financial demands through continuing existing employment [36].

Consistent with the Australian Universities Accord Final Report [9], the Australian Government may be looking to explore novel approaches to manage inequities of access to higher education health courses in rural communities, drawing from local and international examples. One international example from the United Kingdom is the National Health Service (NHS) degree apprenticeship program where nursing and allied health students can earn an income while studying by working part-time in junior or assistant roles [37]. In this approach, the NHS Trust works in partnership with a local university, which enables students to attend university classes two days per week and work three days per week for the Trust. For allied health disciplines, the program length is four years compared to the traditional three years full-time course in the United Kingdom.

Another example of a strategy to address higher education access inequity is the currently expanding Australian ‘Regional University Study Hub’ (RUSH) program [38], formerly known as the ‘Regional University Centres’ (RUCs) program. Funded by the Australian Government since 2018, RUSHs are physical hubs that tertiary students can access to study in courses delivered online by any university across Australia. These study hubs are learning centres with study facilities, equipped with computers and high-speed internet, and provide academic and pastoral support. There is currently a network of 46 RUSHs distributed around Australia [38].

One successful RUSH is in Geraldton, Western Australia, where a collaboration between the Geraldton Universities Centre (GUC) and the University of Southern Queensland has sought to respond to a community’s rural health workforce need. Since 2014, students located in the mid-west of Western Australia have been enrolled as external students in the Bachelor of Nursing program at the University of Southern Queensland, with access to their online learning platform. While formally enrolled as ‘external’ students, they benefit from attending face-to-face tutorials at the GUC facility, led by industry experts. Students also benefit from the other supports provided, such as assistance with administration and enrolment, access to tailored academic learning support, and the opportunity to attend a graduation ceremony locally. The students also undertake their nursing practical placements in the local community, as well their clinical residential schools. Without the GUC, they would have to travel to Queensland to complete placements and intensives. The GUC also has an Australian Nursing and Midwifery Accreditation Council accredited nursing lab, with simulation manikins and associated equipment. Recently, 20 nurses graduated and 19 of these were employed locally. Since the start of the partnership, approximately 152 students have successfully graduated as registered nurses, many of whom still work locally.

In locations where students may not have access to a regional university campus or a RUSH, communities and health services could explore establishing support for health students within their community by collaborating with local libraries, vocational education and training providers, schools and even community/neighbourhood houses [39]. Access to technology and internet connectivity is vital for students to access higher education and therefore developing collaborations to improve their access, as well as support, where they work and live would be beneficial.

Since conducting the audit of course offerings for this study, new allied health courses have commenced or will commence soon for occupational therapy, physiotherapy and speech pathology. Flinders University commenced courses at Port Pirie, Renmark and Mt Gambier Campuses in 2024 for occupational therapy, speech pathology and physiotherapy (https://www.flinders.edu.au/study/health/regional-allied-health-courses). Deakin University has commenced an occupational therapy course at their Warrnambool campus (https://www.deakin.edu.au/course/bachelor-occupational-therapy) and the Australian Catholic University has commenced occupational therapy at the Ballarat campus (https://www.acu.edu.au/study-at-acu/find-a-course/new-courses/bachelor-of-occupational-therapy-ballarat). The University of South Australia is now offering physiotherapy and occupational therapy at their Whyalla campus (https://www.unisa.edu.au/connect/unisa-regional/whyalla-campus/). The University of Tasmania is also commencing an occupational therapy course at their Launceston campus (https://www.utas.edu.au/study/postgraduate/allied-health), increasing access for students in Tasmania where there had not been an occupational therapy course despite long standing workforce shortages. Even though there are new offerings in some rural areas of Australia, more work is required to ensure higher education health courses are accessible for people living rurally.

Another finding from the audit is the issue of inconsistent terminology on university websites can create confusion and mislead prospective students. While the TEQSA Domain 7 of the Higher Education Standards Framework [40] requires universities to clearly articulate how courses are delivered, the audit found that this did not always occur. Some courses were advertised as online although they had additional requirements, such as participation in multiple face-to-face intensive programs. Other inconsistencies identified in the audit included limited information regarding courses availability on university websites, and difficulty determining part-time availability, or part-time availability only being available in early course years. Inconsistent terminology used to describe the nature of involvement in courses may confuse or miscommunicate what is expected of students. Rural students may use the information to make decisions about participation, and if the course requires residency schools not clearly articulated in the course information, they may start the course then withdraw after significant investment and subsequently not join the rural health workforce.

Limitations of this study included the possible lag in professional registration boards and associations posting or updating information on their websites relating to new courses or those being discontinued. To limit the likelihood of missing courses on offer for 2023, we checked university websites to identify current courses and those being discontinued; however, we were reliant on the accuracy of these websites. Although we initially audited the list of approved programs of study by the Psychology Board of Australia according to their website, given the large number of pathway courses for psychology, there may have been some courses included that may not be offered or offered using a different title leading to some inaccuracies.

## Conclusions

The findings of this audit highlight the deficiency of health course availability in Australian regional, rural and remote areas, particularly for some allied health disciplines, dentistry and medicine. Although there has been a recent increase in regional and rural course offerings for some disciplines such as occupational therapy and nursing, rural students are usually required to move or travel large distances to a large regional centre or metropolitan centre to study. To further reduce the need for rural students to relocate or travel to undertake health courses, universities need to increase the flexibility of health course delivery so that students, particularly mature-aged students, can study part-time or via predominantly online mode with any experiential on-campus learning organised in well-funded residential blocks to support rural students to attend. More detailed and clearer course information is needed on university websites to ensure prospective students are making informed decisions regarding their course choice and students can make the necessary arrangements to meet their study commitments. The issues associated with undertaking practice placements such as financial, housing, and managing other non-university commitments of students is an area needing further research, particularly to explore and determine the impact of novel course delivery strategies on access to higher education health courses for rural people.

### Electronic supplementary material

Below is the link to the electronic supplementary material.

Supplementary Material 1

## Data Availability

All data generated or analysed during this study are included in this published article [and its supplementary information files].

## Abbreviations

MMM: Modified Monash Model
MM1: metropolitan areas
MM2: regional centres
MM3: large rural towns
MM4: medium rural towns
MM5: small rural towns
MM6: remote communities
MM7: very remote communities
AHPRA: Australian Health Practitioner Regulation Agency
TEQSA: Tertiary Education Quality and Standards Agency
NDIS: National Disability Insurance Scheme
RUSH: Regional University Study Hub
RUC: Regional University Centre
GUC: Geraldton Universities Centre
