# Supplementary material for: Location and access to health courses for rural students: an Australian audit
Source: BMC Med Educ. 2024 Jul 29;24:806. doi: 10.1186/s12909-024-05787-3 (PMC11285131; doi:10.1186/s12909-024-05787-3)
Supplement: Supplementary file 1 — Supplementary Material 1 [file 12909_2024_5787_MOESM1_ESM.pdf]

Table 1. Nursing/Midwifery courses according to MM location, university, and mode of delivery.

MM	Course	University/Location	Mode of Delivery	Additional Details
**MM2**	Bachelor of Nursing*	Australian Catholic University / Ballarat	Multi-modal	
		Central Queensland University / Rockhampton	Not described	Part-time available.
		Charles Darwin University / Darwin	Not described	Part-time available.
		Charles Sturt University / Albury-Wodonga	Internal	
		Federation University / Mount Helen	Multi-modal	
		La Trobe University / Bendigo	Internal	
		La Trobe University/ Albury-Wodonga	Internal	
		University of Southern Queensland / Toowoomba	Not described	Part-time available.
		University of Tasmania / Launceston	Multi-modal	Part-time available.
		University of Tasmania / Hobart	Multi-modal	Part-time available.
		Charles Sturt University / Albury-Wodonga	Multi-modal	Online with residential schools required for some online subjects.
	Bachelor of Nursing (enrolled nurses)	Australian Catholic University / Ballarat	Multi-modal	
		La Trobe University / Bendigo	On-campus	
		La Trobe University/Albury-Wodonga	On-campus	
	Bachelor of Nursing (graduate entry)	La Trobe University / Bendigo	On-campus	
		La Trobe University / Albury-Wodonga	On-campus	
	Bachelor of Nursing (flexible)	Federation University / Mount Helen	Multi-modal	
	Bachelor of Science	James Cook University /	Not described.	Part-time available.

	Nursing*	Townsville		
		James Cook University / Cairns	Not described	Part-time available.
		James Cook University/Mackay	Not described	
	Bachelor of Science (Nursing)	Edith Cowan University / Bunbury	Internal	Part-time available.
		University of Southern Cross / Hervey Bay	Multi-modal	Part-time available.
	Bachelor of Midwifery	Charles Darwin University / Darwin	Not described	Part-time available.
	Bachelor of Nursing / Bach of Midwifery	La Trobe University / Bendigo	Internal	
	Bachelor of Science (Nursing) / Bach of Science (Midwifery)	Edith Cowan University / Bunbury	Internal	
	Bachelor of Nursing Science / Bach of Midwifery*	James Cook University / Townsville		Part-time available in first year.
	Diploma of Health Care / Bachelor of Nursing	Charles Darwin University / Darwin	Internal	Part time available.
	Bachelor of Nursing / Bachelor of Paramedicine	Federation / Ballarat	Multi-modal	
	Master of Nursing Practice (pre-registration)	Charles Darwin University / Darwin	Internal	Part-time available.
**MM3**	Bachelor of Nursing*	Charles Sturt University/Bathurst	Internal	On-campus, online
		Charles Sturt University / Port Macquarie	Internal	On-campus, online
		Charles Sturt University / Dubbo	Internal	On-campus, online
		Charles Sturt University / Wagga Wagga	Internal	On-campus, online
		Deakin University /	Internal	On-campus

	Warrnambool		
	Federation University/ Churchill	Multi-modal	On-campus, online
	Federation University / Churchill	Multi-modal	Flexible delivery predominantly online and on-campus for intensive blocks each semester.
	La Trobe University / Mildura	Internal	
	La Trobe University/ Shepparton	Internal	
	Southern Cross University / Lismore	Not described	On-campus and online
	Southern Cross University / Coffs Harbour	Not described	On-campus and online
	University of New England / Armidale	Internal	On-campus with some online.
	University of South Australia / Mount Gambier	Multi-modal	On-campus / online. Part-time available.
	University of South Australia / Whyalla	Multi-modal	Part-time available
	University of Tasmania / Launceston	Multi-modal	Part-time available.
	University of Tasmania / Burnie	Multi-modal	Part-time available.
	University of Wollongong / Shoalhaven	Internal	
Bachelor of Nursing (Enrolled nurses)	La Trobe University / Mildura	Internal	
	La Trobe University/ Shepparton	Internal	
	Southern Cross University/ Lismore	Not described	Online and intensive on-campus classes.
	Southern Cross University / Coffs Harbour	Not described	Online and intensive on-campus classes.
Bachelor of Nursing (graduate entry)	La Trobe University / Mildura	Internal	
	La Trobe University/ Shepparton	Internal	
Bachelor of Nursing Science	University of Sunshine Coast / Gympie	Multi-modal	Part-time available.

	Bachelor of Midwifery	University of South Australia / Mount Gambier	Multi-modal	Part-time available.<br>On-campus and online.
**MM4**	Bachelor of Nursing	Federation University / Churchill	Multi-modal	Flexible delivery predominantly online and on-campus for intensive blocks each semester.
		University of Wollongong / Batemans Bay	Internal	
**MM5**	Bachelor of Nursing*	Flinders University / Renmark	Internal	Part-time available.
		University of Wollongong / Bega	Internal	
	Bachelor of Nursing (Enrolled nurses)	University of Notre Dame / Broome	Internal	
**MM6**				
**MM7**	Bachelor of Science Nursing*	James Cook University / Mt Isa	Not described.	
**Online**	Bachelor of Nursing*	Charles Sturt University	External	Online
		Uni of New England	External	Online but with mandatory on-campus intensive schools
		University of Sunshine Coast	External	
		Central Queensland	External	Online but with residential schools
		Charles Darwin University	External	Distance education with some on-campus attendance.<br>Part-time available.
		University of Southern Queensland	External	Flexible delivery but with intensive residential schools
	Bachelor of Nursing Science*	James Cook University	External	Part-time available.
	Diploma of Health Care / Bachelor of Nursing	Charles Darwin University	External	Part time available.
	Bachelor of Midwifery*	Charles Darwin University	External	Distance education with some on-campus attendance.<br>Part-time available.
		University of Sunshine Coast	External	Part time available
	Bachelor of Midwifery	Central Queensland	External	Online but with compulsory residential schools.

	(graduate entry)			
	Master of Nursing Practice (Pre-registration)	Charles Darwin University	External	Online but with on-campus block simulation classes.

*Honours also offered

Table 2. Psychology courses according to MM location, university, and mode of delivery.

MM	Course	University/Location	Mode of Delivery	Additional Details
**MM2**	Bachelor of Psychological Science*	Central Queensland University/Bundaberg, Cairns, Rockhampton, Townsville	Internal	Part time available
		Charles Darwin University/Brinkin	Internal	Part time available
	Bachelor of Science (Psychology)	Central Queensland University/Bundaberg, Cairns, Rockhampton, Townsville	Internal	Part-time available
	Master of Clinical Psychology	Central Queensland University/Rockhampton	Internal	Part-time available
	Bachelor of Psychological Science (grad entry)	Charles Darwin University/Brinkin	Internal	Part-time available
	Bachelor of Psychological Science*	Federation University/Mount Helen	Internal	
	Bachelor of Business/Bachelor of Psychological Science	James Cook University / Townsville	On-campus	
	Bachelor of Psychological Science*	James Cook University / Townsville	On-campus	
	Bachelor of Sport & Exercise / Bachelor of Psychological Science*	James Cook University / Townsville	On-campus	
	Graduate Diploma of Psychology	James Cook University / Townsville	On-campus	
	Master of Psychology (Clinical)	James Cook University / Townsville	On-campus	

Bachelor of Criminology / Bachelor of Psych Science	La Trobe University / Bendigo	On-campus
Bachelor of Psychological Science	La Trobe University / Albury-Wodonga	On-campus
Bachelor of Psychological Science	La Trobe University / Bendigo	On-campus
Bachelor of Arts and Bachelor of Science (Psychology)	University of Southern Queensland / Toowoomba	On-campus
Bachelor of Business ~~and Commerce~~ and Bachelor of Science	University of Southern Queensland / Toowoomba	On-campus
Bachelor of Science* Major Psychology	University of Southern Queensland / Toowoomba	On-campus
Bachelor of Arts*	University of Tasmania / Launceston	
Bachelor of Arts	University of Tasmania/Hobart	
Bachelor of Arts	University of Tasmania / Launceston	
Bachelor of Arts and Bachelor of Business	University of Tasmania / Launceston	
Bachelor of Behavioural Science	University of Tasmania / Launceston	
Bachelor of Psychological Science*	University of Tasmania / Launceston	On-campus
Bachelor of Psychological Science *	University of Tasmania / Hobart	On-campus
Bachelor of Psychological Science	University of Tasmania / Launceston	On-campus
Bachelor of Psychological Science (53F)	University of Tasmania / Hobart	On-campus
Bachelor of Psychology*	University of Tasmania / Launceston	
Bachelor of	University of Tasmania /	

	Science	Launceston		
	Master of Professional Psychology	University of Tasmania / Hobart	On-campus	
	Master of Psychology (Clinical)	University of Tasmania / Hobart	On-campus	
**MM3**	Bachelor of Psychology*	Charles Sturt University/Bathurst NSW, Port Macquarie	Internal	Part-time available
	Bachelor of Social Science (Psychology)*	Charles Sturt University/Bathurst	Internal	Part-time available
	Bachelor of Nursing / Bach of Psychological Science	Deakin/Warrnambool	internal	
	Bachelor of Psychological Science*	Deakin/Warrnambool	Internal	Part-time available
	Bachelor of Psychological Science	Federation University /Churchill	Internal	
	Bachelor of Exercise Science and Psychological Science	Southern Cross University / Coffs Harbour	On-campus	
	Bachelor of Exercise Science and Psychological Science	Southern Cross University / Lismore	On-campus	
	Bachelor of Psychological Science*	Southern Cross University / Coffs Harbour	On-campus	
	Bachelor of Psychological Science/Bachelor of Laws	Southern Cross University / Coffs Harbour	On-campus	
	Bachelor of Arts*	University of New England / Armidale	On-campus	
	Bachelor of Arts / Bachelor of Business	University of New England / Armidale		
	Bachelor of Arts / Bachelor of Laws	University of New England / Armidale		
	Bachelor of	University of New		

	Arts / Bachelor of Science	England / Armidale		
	Bachelor of Psychological Science	University of New England / Armidale	On-campus	
	Bachelor of Psychology*	University of New England / Armidale	On-campus	
	Bachelor of Science*	University of New England / Armidale	On-campus	
	Bachelor of Social Science*	University of New England / Armidale	On-campus	
	Doctor of Philosophy (Clinical Psychology)	University of New England / Armidale	On-campus	
	Graduate Diploma in Psychology	University of New England / Armidale	On-campus	
	Master of Psychology (Clinical)	University of New England / Armidale	On-campus	
	Bachelor of Psychological Science*	Federation University / Churchill	On-campus	
**No courses in MM4 -MM7 were listed on the AHPRA website.**				
**Online**	Bachelor of Psychological Science*	Central Queensland University/ Online	External	Part time available
	Bachelor of Psychological Science	Charles Darwin University/ online	External	
	Bachelor of Psych Science*	Deakin/online	External	Part-time available
	Bachelor of Science (Psychology)	Central Queensland University/Online	External	Part-time available
	Bachelor of Psychological Science (grad entry)	Charles Darwin University/ online	External	Part-time available
	Bachelor of Psychological Science and Criminology	Australian College of Applied Psychology	External	Part-time available. On campus and multi-modal mode options available.
	Graduate	Australian College of	External	Part-time available.

Diploma of Psychological Science	Applied Psychology		
Graduate Diploma of Psychological Science	Cairnmillar Institute/online	External	Part-time available
Graduate Diploma of Psychological Science	Deakin University/online	External	Part-time available
Graduate Diploma of Psychological Science (Advanced)	Deakin University/online	External	Part-time available
Bachelor of Laws/Bachelor of Science (Psychology)	Central Queensland University/online	External	Part-time available
Bachelor of Science (Criminology & Psychology)	Central Queensland University/online	External	Part-time available
Master of Professional Psychology	Central Queensland University/online	External	Part-time available
Master of Clinical Psychology	Charles Sturt University/online	Multi-modal	Residential Schools required for some online subjects. Part-time available.
Master of Professional Psychology	Charles Sturt University/online	Multi-modal	Residential Schools required for some online subjects. Part-time available.
Graduate Diploma of Psychology	Charles Sturt University/online	External	Part-time available
Master of Psychological Practice (with specialisations)	Charles Sturt University/online	Multi-modal	Residential Schools required for some online subjects. Part-time available.
Doctor of Philosophy - Psychology	Curtin University/online	External	
Bachelor of Arts (Psychology)*	Deakin University/online	External	Part-time available
Bachelor of Criminology / Bachelor of Psychological	Deakin University/online	External	Part-time available

Science			
Bachelor of Health Sciences	Deakin University/online	External	Part-time available
Bach of Marketing (Psychology)	Deakin University/online	External	Part-time available
Bachelor of Psychology*	Deakin University/online	External	Part-time available
Bachelor of Psychology	Edith Cowan University/online	External	Part-time available
Bachelor of Psychology*	CSU/online	External	Part-time available
Bachelor of Psychology, Criminology and Justice	Edith Cowan University/online	External	Part-time available
Bachelor of Psychology and Counselling	Edith Cowan University/online	External	Part-time available
Bachelor of Commerce / Bachelor of Psychology	Edith Cowan University/online	External	Part-time available
Bachelor of Laws / Bachelor of Psychology	Edith Cowan University/online	External	Part-time available
Bachelor of Social Science (Psychology)*	Charles Sturt University/ online	External	Part-time available
Graduate Diploma of Psychology (Bridging)	James Cook University / Online	Online	
Bachelor of Psychological Science	La Ttrobe University / Online	Online	
Bachelor of Psychology*	La Trobe University / Online	Online	
Bachelor of Arts – Psychology (OUA)	Macquarie University / Online		
Graduate Diploma in Psychology	Monash University / Online	Online	
Graduate Diploma of	Monash University / Online	Online	

Psychology Advanced		
Bachelor of Psychological Science	Southern Cross University / Online	Online
Bachelor of Psychological Science/ Bachelor of Laws	Southern Cross University / Online	Online
Bachelor of Behavioural Studies (Psychology)	Swinburne University/ Open Universities Australia	Online
Bachelor of Psychological Sciences	Swinburne University/ Open Universities Australia	Online
Bachelor of Psychological Sciences	Swinburne University / Online	Online
Graduate Diploma of Psychology	Swinburne University / Open Universities Australia	Online
Graduate Diploma of Psychology	Swinburne University / Online	Online
Graduate Diploma of Psychology (Advanced)	Swinburne University / Online	Online
Bachelor of Arts*	University of New England / Online	Online
Bachelor of Arts / Bachelor of Business	University of New England / Online	Online
Bachelor of Arts / Bachelor of Laws	University of New England / Online	Online
Bachelor of Arts / Bachelor of Science	University of New England / Online	Online
Bachelor of Audiometry	University of New England / Online	
Bachelor of Psych Science	University of New England / Online	Online
Bachelor of Psychology*	University of New England / Online	Online
Bachelor of Science*	University of New England / Online	Online
Bachelor of Social	University of New England / Online	Online

Science*		
Doctor of Philosophy (Clinical Psychology)	University of New England / Online	Online
Graduate Diploma in Psychology	University of New England / Online	Online
Graduate Diploma in Psychology (Advanced)	University of New England / Online	Online
Master of Professional Psychology	University of New England / Online	Online
Master of Psychology (Clinical)	University of New England / Online	Online
Graduate Diploma in Psychology	University of New South Wales / Online	Online
Bachelor of Psychology	University of South Australia / Online	Online
Bachelor of Arts and Bachelor of Science (Psychology)	University of Southern Queensland / Online	External
Bachelor of Psychology*	University of Southern Queensland / Online	Online
Bachelor of Science* Major Psychology	University of Southern Queensland / Online	External Online
Bachelor of Science Major Psychology Extended	University of Southern Queensland / Online	External Online
Master of Clinical Psychology (Advanced Entry)	University of Southern Queensland / Online	External
Master of Clinical Psychology	University of Southern Queensland /	Online External
Master of Professional Psychology	University of Southern Queensland /	Online External
Bachelor of Psychological Science (53F)	University of Tasmania	Distance - Hobart

Bachelor of Psychological Science and Criminology	Western Sydney University / Online	Online
Bachelor of Psychological and social sciences	Western Sydney University / Online	Online
Bachelor of Business/Bach of Psychological Science	James Cook University / Online	External
Graduate Diploma of Psychology	James Cook University / Online	External

*Honours also offered
